# Trajectory of Psychological Adaptation During an 8-Day Fast: Changes in Subjective Well-Being, Mood, and Neuroendocrine Responses

**DOI:** 10.3390/nu18213634

**Published:** 2026-10-31

**Authors:** Karol Pilis, Krzysztof Stec, Wiesław Pilis, Alicja Żak, Anna Pilis, Paweł Dolibog, Alicja Minto

**Affiliations:** 1 Collegium Medicum, Jan Dlugosz University in Czestochowa, 42-200 Czestochowa, Poland; k.pilis@ujd.edu.pl (K.P.); w.pilis@ujd.edu.pl (W.P.); a.pilis@ujd.edu.pl (A.P.); 2 Department of Economy in Opole, WSB Merito University in Wroclaw, 53-609 Wrocław, Poland; alicja.zak@opole.merito.pl (A.Ż.); alicja.minto@opole.merito.pl (A.M.); 3 Department of Biophysics, Faculty of Medical Sciences in Zabrze, Medical University of Silesia, 40-055 Katowice, Poland; pawel.dolibog@sum.edu.pl

**Keywords:** mood, metabolic stress, psychological adaptation, serotonin, opioid peptides, cortisol

## Abstract

Background/Objectives: Prolonged fasting induces substantial metabolic and neuroendocrine adaptations; however, less is known about the accompanying psychological changes. This study evaluated changes in subjective well-being and mood during an 8-day fast in relation to serum concentrations of α -endorphin, serotonin, oxytocin, and cortisol. Methods: Fifteen male subjects in good health completed an 8-day fast. Subjective well-being was assessed each day using a comparative self-rating scale, and mood was evaluated using the Polish version of the UWIST Mood Adjective Checklist (UMACL). Serum concentrations of α-endorphin, serotonin, oxytocin, and cortisol were measured before and after the fast, both at rest and following a maximal exercise test on an ergometer. Results: Subjective well-being gradually improved during the fasting period, and tense arousal, a dimension of mood, decreased. No significant changes were observed in energetic arousal or hedonic tone. The combination of fasting and exercise was associated with significant increases in α-endorphin, serotonin, and cortisol concentrations. Fasting was associated with a significant increase in post-exercise cortisol concentrations, whereas exercise after fasting was associated with a significant increase in serotonin concentrations. Despite concurrent psychological and hormonal adaptations, no consistent relationships were found between changes in hormone concentrations and psychological variables. Conclusions: Prolonged fasting was accompanied by gradual psychological adaptation, reflected in improved subjective well-being and reduced tension, whereas exercise under these conditions was associated with neurohormonal changes. These changes were associated with the coordinated activation of multiple neuroendocrine systems, suggesting that psychological and neuroendocrine adaptations represent complementary responses to prolonged metabolic stress rather than being opposing processes.

## 1. Introduction

Prolonged fasting induces complex metabolic, neuroendocrine, and psychological changes as part of the body’s adaptation to reduced energy intake [1]. In addition to its well-documented physiological consequences, the effects of fasting on psychological functioning, including current well-being, mood, and broader subjective well-being, have attracted increasing interest [1,2,3,4,5,6]. Findings to date have been mixed: improvements in selected aspects of psychological functioning have been reported alongside increased psychological stress or no significant changes in mood. This suggests that the psychological response depends on the type and duration of the fasting intervention and the population studied. A better understanding of the mechanisms of psychological adaptation to prolonged fasting therefore remains an important research objective.

Contemporary psychological theories regard affect as a fundamental dimension of human emotional functioning, encompassing brief emotions, more enduring affective states, and cognitive evaluations of one’s life [7]. Although mood and subjective well-being are related, they are distinct constructs in psychology and biomedicine [8]. Mood is defined as a relatively enduring affective state of moderate intensity that, unlike emotion, need not be directly linked to a clearly identifiable stimulus or object [9,10]. The theoretical model proposed by Matthews et al. [10] describes a three-factor structure of mood comprising energetic arousal, tense arousal, and hedonic tone. These dimensions are conceptually distinct but empirically related. Energetic arousal refers to vitality and energy, tense arousal to tension and anxiety, and hedonic tone to the valence of affective experience. The authors also showed that energetic arousal and tense arousal are not opposite ends of a single continuum but separate factors that can vary independently. This account is consistent with the concept of core affect, in which basic emotional experience can be described along two dimensions: valence (pleasure–displeasure) and activation (arousal–deactivation) [7].

Subjective well-being is a broader construct than mood. It includes an affective component, reflecting the frequency and intensity of positive and negative states, and a cognitive component, reflecting an overall appraisal of one’s functioning and quality of life [8]. In contrast to multidimensional instruments assessing global well-being, intervention studies increasingly use brief, single-item measures of current subjective well-being to capture day-to-day changes in how individuals perceive their condition. This approach allows fluctuations in well-being to be assessed through repeated measurements, and responses to be anchored to each individual’s usual state, thereby reducing the influence of between-person differences [11].

Well-being and mood are regulated by multiple neurobiological mechanisms involving interactions among the nervous, endocrine, and metabolic systems. Fasting is a particularly potent stimulus for these mechanisms and induces a range of adaptive changes in bodily functioning.

Fasting imposes substantial metabolic stress and initiates adaptive mechanisms, including activation of the hypothalamic–pituitary–adrenal (HPA) axis and modulation of neurotransmitter systems involved in the regulation of stress responses and affect. In their literature review, Fond et al. [1] describe fasting as a potent physiological stimulus that influences neurobiological mechanisms involved in mood regulation.

Empirical studies of the effects of fasting on affective functioning have yielded variable results, potentially reflecting differences in fasting protocols, study populations, and psychometric instruments. In an experimental study of a 48 h zero calorie fast in amateur weight lifters, Solianik et al. [6] reported a significant increase in anger, whereas the other mood states assessed did not change significantly.

In a study of an 8-day fast in middle-aged men, Stec et al. [3] primarily assessed stress and anxiety (PSS, STAI). They reported favorable physiological changes without deterioration in psychological well-being on the measures assessed.

In a clinical population, Jeitler et al. [4] conducted a randomized controlled trial in which a 5 day very low calorie fast preceded a multimodal lifestyle intervention in patients with metabolic syndrome. Depression and fatigue subscale scores were significantly lower in the first week, and vigor was higher at week 12, compared with the control group.

Nugraha et al. [5] reported reduced fatigue and depressive symptom severity during prolonged Ramadan fasting in healthy young adults. The regulation of well-being and mood involves complex interactions among the nervous system, endocrine system, and metabolism. Serotonin, opioid peptides, oxytocin, and cortisol have particular roles in these interactions, participating in responses to metabolic stress and exercise as well as in the regulation of emotional processes and adaptive mechanisms. Previous studies suggest that fasting and exercise can substantially influence the activity of these systems, although the direction and magnitude of the observed changes remain unclear.

Serotonin is a major neurotransmitter involved in mood regulation. It contributes to the regulation of emotional processes, cognitive function, and adaptive mechanisms and is an important component of the gut–brain axis, linking central nervous system function with metabolic processes in the gastrointestinal tract [12,13,14,15]. Previous research suggests that fasting and exercise can influence serotonin concentrations, but findings have been inconsistent. Reduced brain serotonin concentrations have been reported during fasting [16], whereas increased blood concentrations have been observed after Ramadan fasting [17] and aerobic training [18]. Conversely, no changes in salivary serotonin concentrations were observed in some runners experiencing a runner’s high [19], suggesting that the serotonergic response depends on the type and conditions of the intervention.

Opioid peptides are also involved in the regulation of mood and well-being. Among these, β-endorphin, the principal endorphin metabolized to α-endorphin and γ-endorphin, has a central role. Both exercise and fasting can increase opioid system activity. Increases in β-endorphin concentrations have been observed after 30 min of exercise at 80–85% of VO_2_max in runners and swimmers [20], in individuals with severe depression undergoing exercise training [21], in pregnant women [22], and during yoga practices in patients with chronic inflammatory diseases and obesity [23]. A 48 h fast has also been shown to increase basal hypothalamic β-endorphin release, influencing HPA axis regulation [24]. Changes in opioid peptide activity have also been observed in the gastrointestinal tract, where these peptides contribute to the regulation of intestinal motility during fasting [25].

Oxytocin is another important regulator of emotional functioning. It is involved in social, reproductive, and emotional behaviors and in appetite regulation [26], although its role in mood modulation remains unclear [27]. Exercise has been shown to increase blood oxytocin concentrations [28], and oxytocin may contribute to the antidepressant and mood-enhancing effects of physical activity, particularly when performed in groups [27]. Conversely, reduced oxytocin concentrations have been observed in women with anorexia nervosa [29] and in individuals with obesity following a very low calorie ketogenic diet [30].

Cortisol is another biomarker involved in mood regulation. As the principal hormone of the HPA axis, it has a central role in the body’s response to stress. Cortisol concentrations vary with the duration of fasting [31]. Exercise also affects cortisol secretion, although the nature of these changes depends on the type and intensity of activity. Reduced cortisol secretion following exercise training has been observed in individuals with recurrent depressive disorders [21]. In healthy young men, intense exercise was also associated with an attenuated stress response and smaller increases in cortisol concentrations [32].

Taken together, these findings indicate that fasting and exercise induce complex and heterogeneous changes in neurohormonal systems involved in the regulation of mood and well-being. However, the extent to which changes in individual biomarkers are associated with concurrent changes in well-being and multiple dimensions of mood during prolonged fasting remains unclear. Most studies have focused on individual indicators of psychological well-being, or on symptoms of depression, anxiety, or stress. Fewer have examined the multidimensional structure of mood and its relationships with neurohormonal changes induced by fasting and/or exercise. Studies using repeated daily measurements to track changes in well-being and mood throughout a fast also remain limited in number.

To address these gaps, the present study aimed to assess changes in well-being and in multiple dimensions of mood during an 8-day fast, and to examine the relationships of such changes with changes in α-endorphin, serotonin, oxytocin, and cortisol concentrations, measured at rest and after exercise, both immediately before and immediately after the period of fasting.

## 2. Materials and Methods

### 2.1. Participants

Initially, 18 subjects volunteered for the study, including 16 males and 2 females. During the study, 3 participants, including both females, chose not to continue fasting, and their measurements were excluded from the final analyses, which were thus based exclusively on the data obtained from the remaining 15 males. These men represented three occupational groups: civil servants, business people, and academic researchers. Fourteen of them had completed higher education.

Eligibility required previous experience of fasting of at least 7 days in length, during which mineral water only had been consumed. Participants in this study were in good, stable physical and psychological condition before and during the period of fasting, and tolerated food restriction well. They underwent a medical examination three days before the fast. Their strong motivation to participate was accompanied by a positive attitude toward undertaking another period of fasting. At the outset, participants expected improvements in health and a reduction in body weight. During the 8-day fast, the subjects continued to live at home, while maintaining their usual lifestyles, and continuing to fulfill their work, family, and social commitments. Throughout the period of fasting and for 14 days thereafter, they remained in telephone contact with the study coordinator, with a physician, and with a psychologist. Information obtained from the subjects during this period indicated that they experienced no cognitive, emotional, or behavioral disturbances. Nor were any somatic complaints identified.

Before the study began, volunteers were informed about the study methods and experimental conditions, with particular attention being paid to safety during and after fasting. Eligibility criteria were (i) previous experience with prolonged fasting; (ii) no dietary restriction in the preceding 6 months; (iii) no smoking, alcohol consumption, or illicit drug use; (iv) no acute or chronic psychiatric or somatic illness; (v) no eating disorders or other eating-related psychological dysfunction; and (vi) no medical contra-indications to an intervention involving an 8-day fast and intense exercise. Participants were informed about the potential adverse health consequences of the experiment, particularly with regard to the fasting intervention.

All subjects provided written informed consent to their participation in the study.

### 2.2. Demographic Measurements

The mean age of the subjects (all male) was 59.8 ± 12.37 years. Detailed somatic and demographic characteristics are presented in Table 1.

More detailed demographic and anthropometric characteristics of this cohort have been reported previously [33].

#### 2.2.1. Assessment of Well-Being

Well-being was assessed on a daily basis throughout the 8-day fast using a single comparative question. Each day, participants answered the question “How do you feel today?” by selecting one of three responses: “worse than usual,” “same as usual,” or “better than usual.” Responses were coded as 1, 2, and 3, respectively.

The question assessed current well-being relative to each participant’s usual level of functioning, rather than a relatively stable level of psychological well-being. Repeated daily measurements allowed within-person changes to be assessed across successive days of fasting.

The procedure followed a daily diary approach, allowing short-term changes in well-being to be monitored during the intervention. Repeated measurements on successive days allowed the direction of change in well-being to be examined across the entire 8-day fast.

For statistical analysis, daily well-being ratings were treated as repeated ordinal observations nested within participants. Given the three-category ordinal response scale (1 = “worse than usual,” 2 = “same as usual,” 3 = “better than usual”), changes in well-being over time were analyzed using a cumulative link mixed model (CLMM) with a logit link. Fasting day was included as a fixed effect and participant as a random effect (random intercept), accounting for the dependence among repeated measurements from the same individual. The model was used to assess the direction of change in the probability of reporting a higher well-being category with each successive day of fasting.

#### 2.2.2. Assessment of Mood

Mood was assessed using Goryńska’s Polish adaptation of the UWIST Mood Adjective Checklist (UMACL) [9,10]. The instrument measures current emotional state and includes three dimensions of mood: tense arousal (TA), hedonic tone (HT), and energetic arousal (EA). Higher scores indicate greater tension, a more positive mood, and greater energy, respectively. The questionnaire has good psychometric properties and is widely used to investigate short-term changes in mood.

Given differences in how mood is defined and distinguished from emotion in the literature, this study adopted the account proposed by Doliński [34]. In this account, mood is an affective state with a particular valence and relatively low intensity that is more enduring and less closely linked to a specific object or event than an emotion. The study examined changes in mood over the course of an 8-day fast, rather than the short-term affective response to a single exercise stimulus. Accordingly, the UMACL was administered once daily under standardized resting conditions. No additional assessment was performed immediately after the exercise test because it could also have captured the acute affective response to exercise, making it difficult to distinguish this response from fasting-related changes in mood. Assessing such a response would also have required consideration of potential confounders, including individual differences in the subjective experience of exercise, which could not be adequately controlled given the study design and small sample. Limiting assessment to once daily also avoided introducing an additional factor associated with repeated questionnaire administration over a short interval. Previous research suggests that repeated administration of self-report measures of mood or affective states can influence subsequent scores, although the direction and magnitude of this effect are unclear [35]. The exercise tests on the first and eighth study days were instead used to assess neurohormonal reactivity.

#### 2.2.3. Exercise Test

Participants arrived at the laboratory between 7:00 and 8:00 a.m. Psychological assessments and measurements of somatic characteristics were performed first. After 10 min of rest, blood was collected from an antecubital vein for hormonal and metabolic assays, followed by measurements of heart rate and blood pressure.

After the resting assessments, participants performed a leg exercise test on a cycle ergometer. The test began at a workload of 30 W, maintained for 3 min at a constant cadence of 60 rpm. The workload was then increased by 30 W every 3 min while the same cadence was maintained, until participants reached their maximal exercise capacity or a test termination criterion was met.

The test was terminated if (i) the participant could not maintain a cadence of 60 rpm; (ii) systolic blood pressure exceeded 220 mmHg; (iii) age-predicted maximal heart rate was reached; or (iv) maximal oxygen uptake (VO_2_max) was reached and subsequently declined despite continued exercise.

Blood was collected again 3 min after the exercise test ended. Samples were allowed to clot for 10 min and then centrifuged for 10 min at 3000 rpm. The resulting serum was stored at −80 °C until biochemical analysis.

#### 2.2.4. Physiological and Biochemical Procedures

Exercise testing was performed on an Excalibur Sport cycle ergometer (Lode, Groningen, The Netherlands). Heart rate and oxygen uptake were recorded using an Ergo-Card analyzer (Medisoft S.A., Sorinnes-Dinant, Belgium).

Serum serotonin, oxytocin, and α-endorphin concentrations were measured by enzyme-linked immunosorbent assay (ELISA, ELK Biotechnology Co., Ltd. Sugar Land, Texas, USA) using a competitive inhibition enzyme immunoassay technique. The Human 5-HT (5-Hydroxytryptamine) ELISA Kit, Human OT (Oxytocin) ELISA Kit, and Human AEP (α-Endorphin) ELISA Kit were used (ELK Biotechnology Co., Ltd., Denver, CO, USA; catalog numbers ELK8524, ELK9544, and ELK2087; sensitivity 4.05 ng/mL, 5.33 pg/mL, 4.45 pg/mL; detection range 14.07–900 ng/mL, 15.63–1000 pg/mL, 15.63–1000 pg/mL, respectively). The tests in question are characterized by high sensitivity and excellent specificity in the detection of human 5-HT, OT, AEP. No significant cross—reactivity or interference was observed between human 5-HT, OT, and AEP and their analogs. Absorbance was measured at 450 nm using an MINDRAY MR96A spectrophotometer (Shenzhen Mindray Bio-Medical Electronics Co., Ltd., Nanshan, Shenzhen, China). The intra-assay coefficient of variation (CV) was below 8% for all assays. The inter-assay coefficient of variation (CV) was below 10% for all assays.

Cortisol concentrations were measured by chemiluminescence immunoassay (CLIA) REF 130298002M using MAGLUMI Cortisol-CLIA kits (Shenzhen New Industries Biomedical Engineering Co., Ltd., Shenzhen, China). Sensitivity 2.5 ng/mL β-Hydroxybutyrate (β-HB) concentrations were also measured using the RANBUT diagnostic kit (Randox Laboratories Ltd., Crumlin, UK). Catalog numbers SKU: RB1007, sensitivity 0.1 mmol/L. All biochemical assays were performed in replicates.

#### 2.2.5. Statistical Analysis

Statistical analyses were performed using Statistica 13.3 (TIBCO Software Inc., Palo Alto, CA, USA) and jamovi (The jamovi project).

Changes in the UMACL mood dimensions (TA, HT, and EA) were analyzed using linear mixed models (LMMs). In each model, measurement day was included as a fixed effect and participant as a random effect (random intercept), accounting for the dependence among repeated observations from the same individual. Measurement day was treated as a continuous variable to assess the linear trend over time. Model results were reported as regression coefficients (β), standard errors (SE), 95% confidence intervals (95% CI), and *p*-values. For each model, marginal R^2^, representing the proportion of variance explained by the fixed effect, and conditional R^2^, accounting for both fixed and random effects, were also reported.

Because well-being was measured using three ordered response categories, changes in well-being were analyzed using a cumulative link mixed model (CLMM), with measurement day as a fixed effect and participant as a random effect (random intercept). Measurement day was treated as a continuous variable to assess the change in the probability of reporting a higher well-being category over time. The model result was reported as an odds ratio (OR) with a 95% confidence interval and *p*-value.

For variables with a significant effect of time in the mixed models, an additional comparison was made between the first and eighth days of fasting. The Wilcoxon signed-rank test was used for well-being because of its ordinal measurement scale. This test was also used for TA because the distribution of differences between the first and eighth measurements deviated from normality. Effect size was calculated as r = |Z|/√N, where N was the number of pairs with nonzero differences. Means and standard deviations, together with medians and quartiles, were reported for the time points compared. No additional comparisons were performed for HT or EA because the mixed models showed no significant effect of time.

Given the small sample and departures from normality in some hormonal variables, differences in hormone concentrations across the four measurement conditions were assessed using the Friedman test for repeated measures. Effect size was assessed using eta squared (η^2^). When the Friedman test was significant, Dunn–Bonferroni post hoc comparisons were performed.

Correlations between psychological variables and hormone concentrations measured before and after fasting were also examined using Spearman’s rank correlation coefficient.

Relationships between changes in psychological variables and changes in neurohormone concentrations were examined using Spearman’s rank correlation coefficient. The Benjamini–Hochberg correction was applied to control the false discovery rate (FDR) arising from multiple testing. Statistical significance was set at *p* < 0.05; for correlation analyses, significance was determined using FDR-adjusted *p*-values.

## 3. Results

The study aimed to characterize participants’ well-being and mood and their changes during an 8-day fast, and to examine relationships between changes in well-being and mood and changes in the neuroendocrine biomarkers assessed.

The linear mixed models showed a significant decrease in TA during the 8-day fast. No significant changes were found in EA or HT. Detailed results are presented in Table 2.

### 3.1. Well-Being

**Figure 1:**
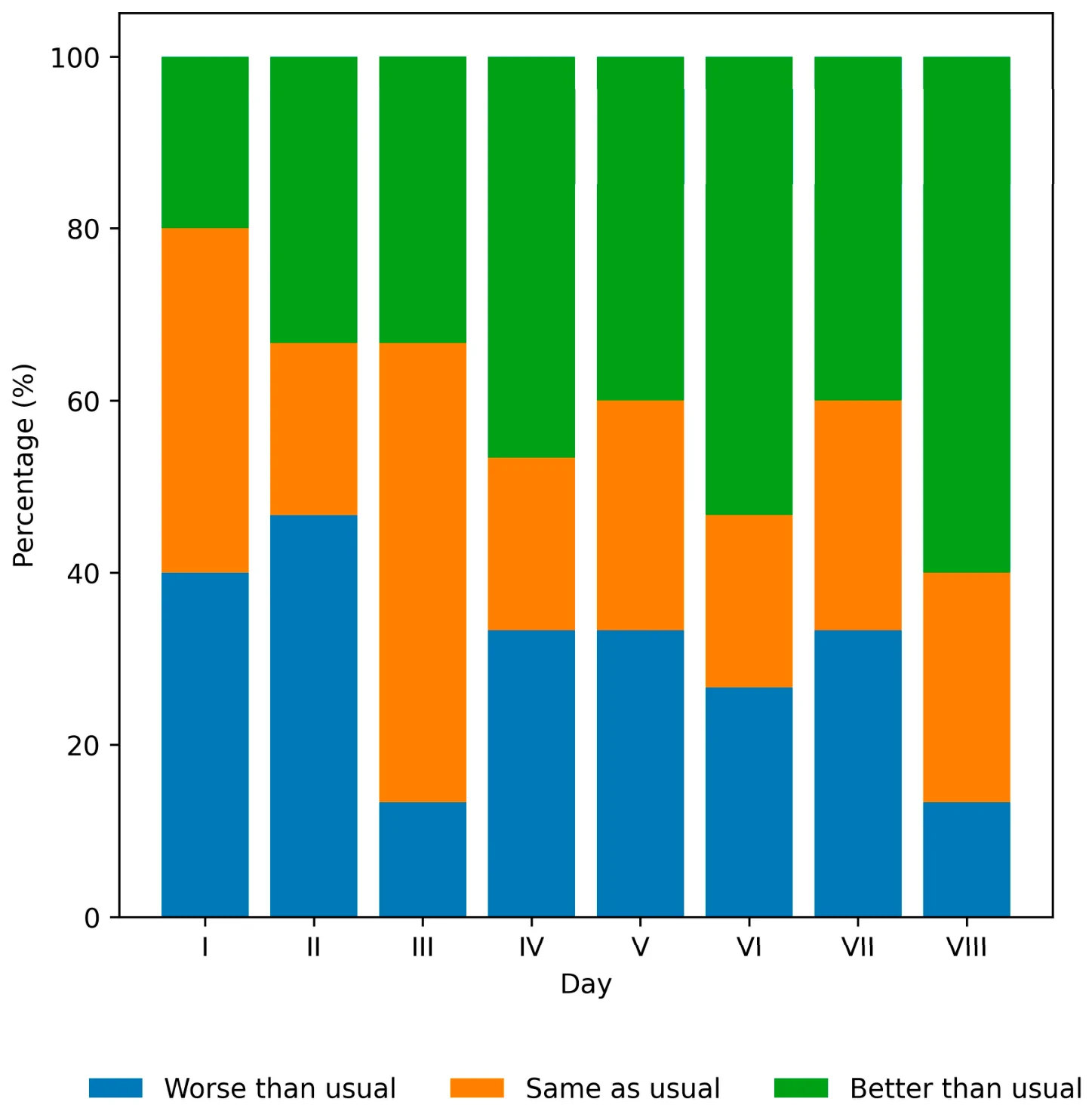
Distribution of subjective well-being responses across the 8-day fasting period. Participants rated their daily well-being as worse than usual, same as usual, or better than usual. Data are presented as percentages. A gradual decrease in “worse than usual” responses and an increase in “better than usual” responses were observed over time, with the highest proportion of positive well-being reported on day VIII.

Given the ordinal measurement of well-being, changes across successive days of fasting were assessed using a cumulative link mixed model (CLMM). The analysis showed a significant effect of time (χ^2^(1) = 6.77; *p* = 0.009). With each successive day of fasting, the odds of reporting a higher well-being category increased by 24.5% (OR = 1.245; 95% CI: 1.055–1.467). Daily well-being ratings are presented in Table 3. The percentage distribution of responses illustrates how well-being varied across days. A clear trend was evident: the number of participants reporting worse well-being than usual decreased, whereas the number reporting better well-being than usual increased as fasting progressed (see Figure 1). The proportion reporting their usual level of well-being also fluctuated. This proportion was highest on the third day, at 53.33%, and lowest on the second, fourth, and sixth days, at 20%. The proportion reporting better well-being than usual increased from 20% on the first day to 60% on the final day.

**Figure 2:**
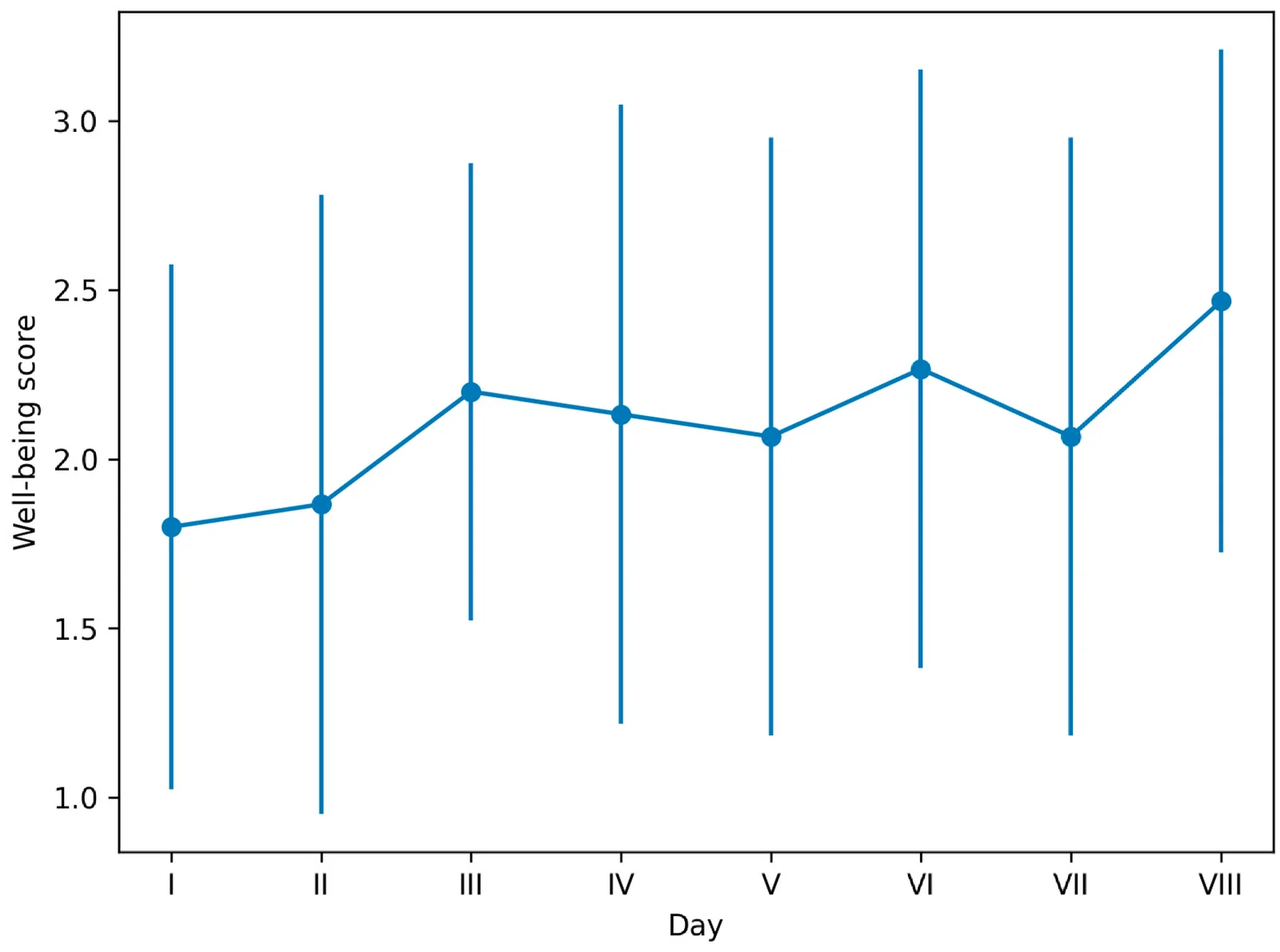
Changes in daily well-being scores during the 8-day fasting period (I–VIII). Data are presented as mean ± SD (n = 15). Well-being was assessed using a 3-point scale (1 = worse than usual, 2 = same as usual, 3 = better than usual).

An analysis of the mean scores for each day also showed a clear upward trend in well-being, with some fluctuations on the fourth, fifth, and seventh days (see Figure 2).

Additional pairwise comparisons using the Wilcoxon signed-rank test showed significantly higher well-being on day VIII than on day I (Table 4).

### 3.2. Mood

**Figure 3:**
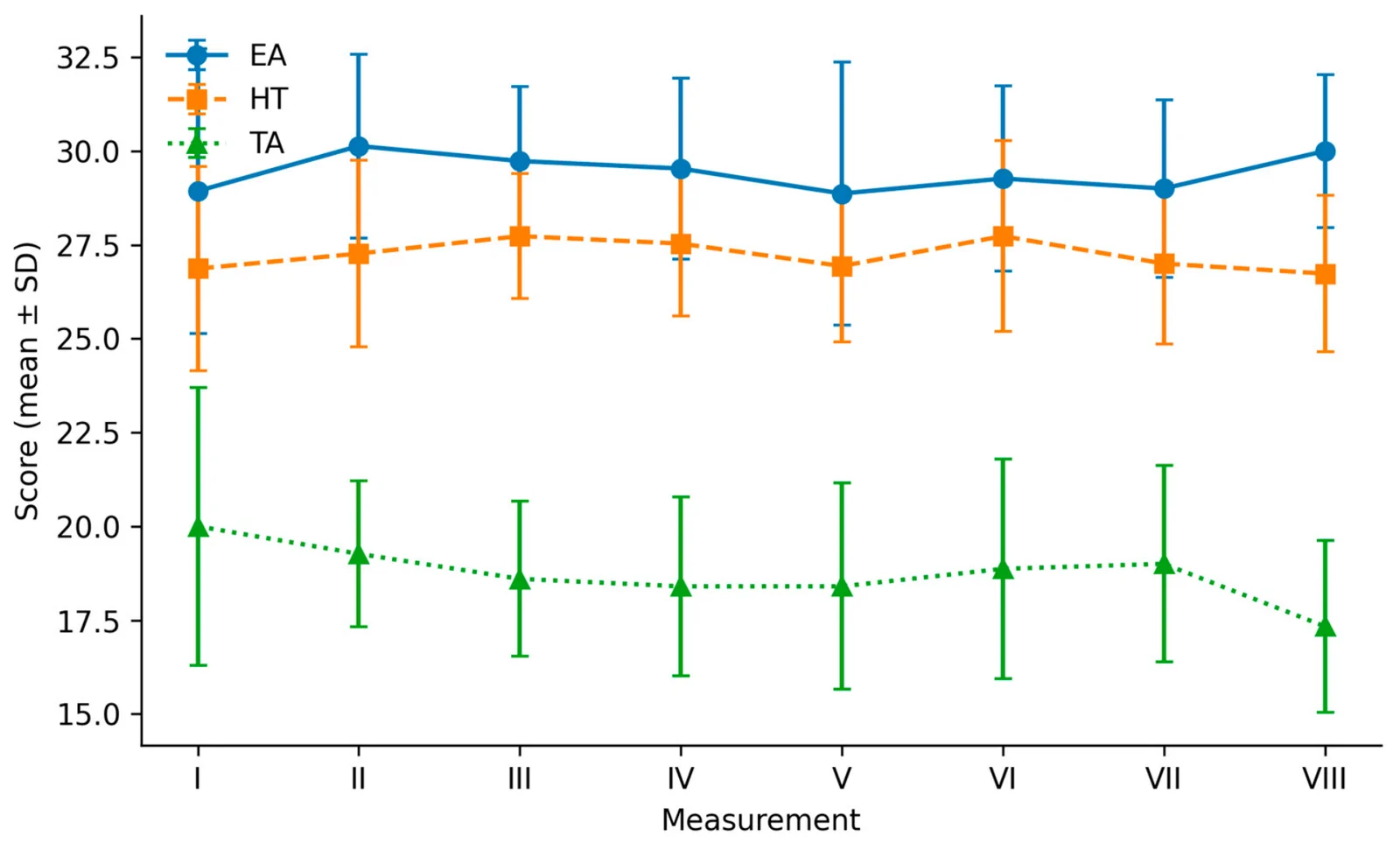
Changes in TA (tense arousal), EA (energetic arousal), and HT (hedonic tone) scores across eight repeated measurements (I–VIII).

Mood was also assessed daily throughout fasting using the UWIST Mood Adjective Checklist (UMACL) [10]. The questionnaire comprises three mood dimensions: tense arousal (TA), energetic arousal (EA), and hedonic tone (HT). Scores for each dimension across successive days of fasting are shown in Figure 3.

#### 3.2.1. Tense Arousal

The linear mixed model showed a significant negative trend in tense arousal over time (Table 2). The fixed effect of time explained 3.8% of the variance in TA (marginal R^2^ = 0.038), whereas the full model, accounting for individual differences among participants, explained 21.7% of the variance in scores (conditional R^2^ = 0.217).

An additional comparison of the first and eighth days of fasting showed significantly lower TA on day VIII than on day I (Table 5).

#### 3.2.2. Energetic Arousal

The linear mixed model showed no significant change in EA during fasting (Table 2). The fixed effect of time explained virtually none of the variance in EA (marginal R^2^ < 0.001), whereas the full model, accounting for individual differences among participants, explained 28.2% of the variance in scores (conditional R^2^ = 0.282).

#### 3.2.3. Hedonic Tone

The linear mixed model showed no significant change during fasting (Table 2). The fixed effect of time explained 0.1% of the variance in HT (marginal R^2^ < 0.001), whereas the full model, accounting for individual differences among participants, explained 37.6% of the variance in scores (conditional R^2^ = 0.376).

### 3.3. Neuroendocrine Markers

The Friedman test showed significant differences in circulating concentrations of α-endorphin, serotonin, and cortisol concentrations across the four measurement conditions: at rest and after exercise, both before and after the 8-day fast. No significant differences in oxytocin concentrations were found across these conditions.

#### Comparisons of Measurements Before and After Fasting

Alpha-endorphin, serotonin, and cortisol concentrations increased significantly following the combination of fasting and exercise. Serum concentrations measured after exercise following the fasting intervention were higher than those measured at rest before the 8-day fast. These differences reached *p* < 0.001 for α-endorphin, serotonin, and cortisol.

Exercise was additionally associated with higher serotonin concentrations, as indicated by a significant difference between resting and post-exercise measurements after fasting (*p*= 0.028), although no such difference was observed before fasting.

Fasting was associated with higher cortisol concentrations, as reflected in the significant difference between post-exercise measurements before and after the fasting intervention (*p* = 0.004), although no such difference was observed under resting conditions.

Detailed comparisons are presented in Table 6.

**Figure 4:**
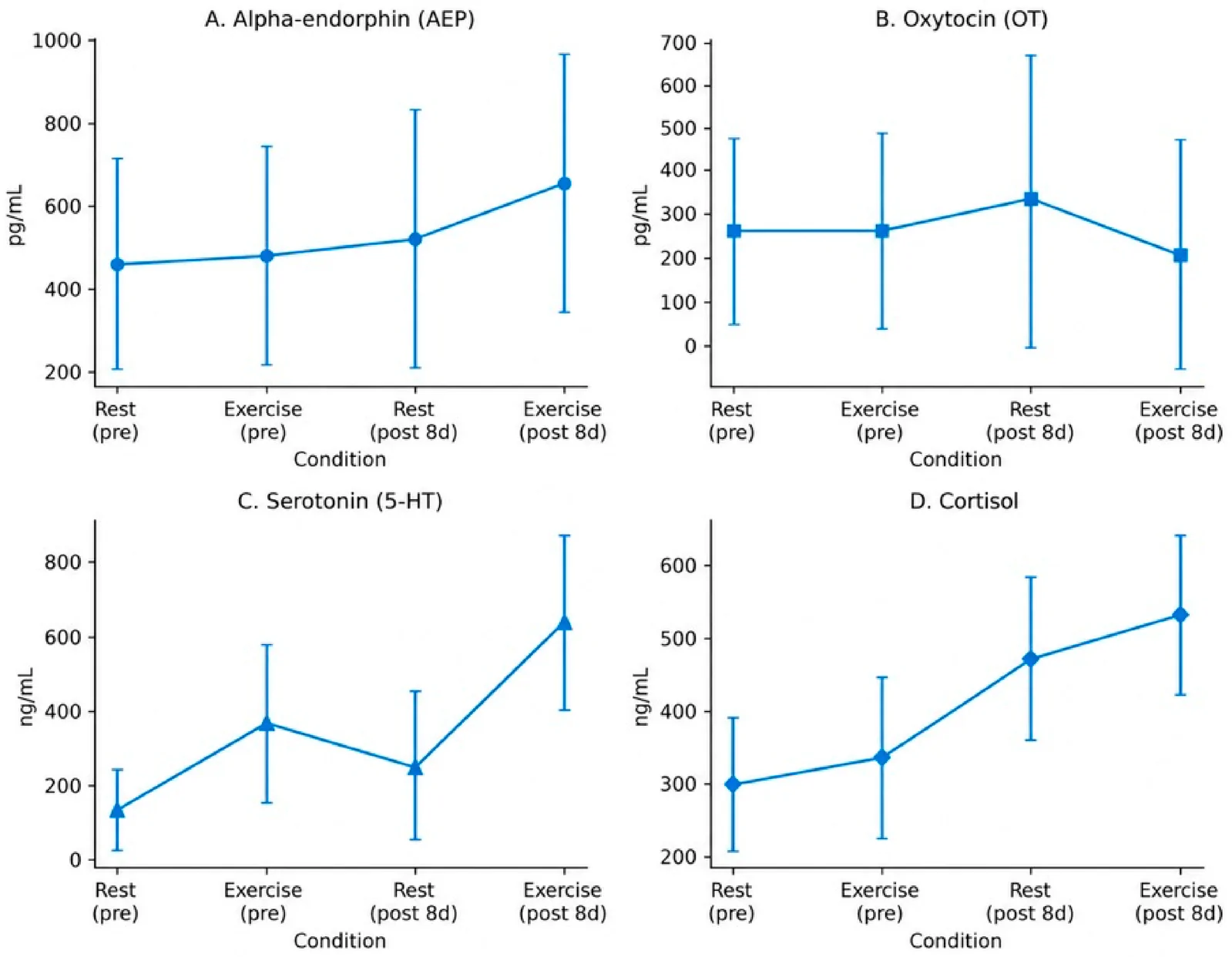
Mean concentrations (± SD) of α-endorphin (AEP), oxytocin (OT), serotonin (5-HT), and cortisol measured at rest and after exercise before and after the 8-day water-only fasting intervention. Error bars represent standard deviations.

Changes in the hormone concentrations assessed are shown in Figure 4. Alpha-endorphin, serotonin, and cortisol concentrations increased, reaching their highest values after exercise performed following 8 days of fasting. Oxytocin concentrations showed no clear direction of change across the measurement conditions.

### 3.4. Relationships of Well-Being and Mood with α-Endorphin, Serotonin, Oxytocin, and Cortisol

#### 3.4.1. Correlations for Measurements Before and After Fasting

Correlation analyses were performed separately for each hormone before and after the 8-day fast. To limit the risk of type I error, the Benjamini–Hochberg FDR correction was applied separately to each hormone type.

Before fasting, several relationships were observed between psychological variables and hormone concentrations. After FDR correction, only two negative correlations remained statistically significant: those of post-exercise serotonin concentrations before fasting with overall well-being and hedonic tone. No significant relationships involving cortisol remained after adjustment for multiple comparisons.

After fasting, none of the relationships of psychological variables with serotonin or cortisol concentrations remained statistically significant following FDR correction. Detailed correlation results are presented in Table 7.

#### 3.4.2. Correlations Between Changes from Before to After Fasting

The analysis of correlations between changes (Δ) in resting hormone concentrations and changes in psychological variables revealed no statistically significant relationships (Table 8).

## 4. Discussion

The 8-day fast was associated with significant changes in the subjects (all male), involving both psychological functioning and neuroendocrine responses. In addition to marked ketosis, confirmed by a significant increase in β-HB concentrations [33], subjective well-being improved and tense arousal decreased, alongside neuroendocrine changes. Despite the concurrent occurrence of these changes, no consistent relationships were found between the psychological and neuroendocrine responses. These findings indicate that adaptation to prolonged fasting is multifactorial and, in addition to the well-documented neuroendocrine mechanisms that enable adaptation to energy deficiency [36], may also involve changes in psychological functioning [1].

### 4.1. Changes in Mood and Well-Being

A major finding was that subjective well-being tended to improve and tense arousal to decrease during the 8-day fast. Daily assessments and mixed models allowed changes throughout fasting to be evaluated, rather than only the difference between states at the beginning and the end of the fasting period. Although some earlier studies also assessed psychological functioning during fasting, they included modified fasting protocols rather than exclusively prolonged fasting [37,38]. The study most similar in terms of protocol to that here under review was conducted by Yang et al. [39]. In that study, during a 10 day fast undertaken by male subjects in good health, it was found that changes in subjective states did not follow a constant, unidirectional trend but, instead, U-shaped or inverted U-shaped trajectories. Likewise, changes in well-being and tense arousal in this current study were not uniform, although their overall direction across the 8-day fast indicated improved well-being and reduced tension. Importantly, it should be noted that the volunteers participating in the study had prior experience with prolonged fasting interventions, which may have enhanced their positive expectations regarding the fast and/or contributed to a placebo effect. This pattern may reflect a dynamic process of psychological adaptation to a prolonged physiological challenge.

The increase in cortisol concentrations observed in this current study is consistent with previous reports indicating that increased HPA axis activity forms part of neuroendocrine adaptation to energy deficiency [36]. Colling et al. [40] reported similar findings during a 10 day fast: both free and total cortisol concentrations increased significantly, with a particularly pronounced increase in free cortisol in men. In the present study, however, increased cortisol concentrations were accompanied by improved subjective well-being and reduced tense arousal, and changes in cortisol concentrations did not correlate significantly with psychological changes. These findings suggest that increased cortisol concentrations during prolonged fasting may form part of the physiological adaptive response to energy deficiency and need not necessarily indicate deterioration in psychological functioning. In the stress response, increased cortisol concentrations may support resource mobilization and processes that enable effective coping with the stressor and restoration of homeostasis. Adverse consequences, by contrast, are associated with situations in which adaptation and coping are unsuccessful [41].

Interpretation of these findings requires consideration of differences in how mood has been operationalized in previous studies. Mood is a relatively enduring affective state, distinct from short-term emotional responses to a specific stimulus [34]. However, some studies of physical activity assessed affective states immediately before and after a single exercise session, primarily capturing its immediate affective effects [42]. In the present study, daily measurements allowed mood changes to be assessed over the entire 8-day fast.

Improved well-being was accompanied only by a reduction in TA, with no significant changes in EA or HT. This pattern suggests that improved well-being was associated primarily with reduced psychological tension, rather than an increase in positive mood tone or subjectively perceived energy. These findings are consistent with the authors’ earlier observations of no deterioration in psychological well-being, assessed using the PSS and STAI, during an 8-day fast [3]. Participants in the present study had prior experience with prolonged fasting and reported a positive attitude toward this intervention, which may have partly influenced their subjective well-being ratings. By contrast, Solianik and Sujeta [43] found that a 48 h fast in individuals without previous fasting experience was associated with greater psychological and physiological stress. Differences between the two studies may therefore reflect participants’ previous experience and preparation for fasting as well as the duration of the fasting period.

Experimental studies suggest that reduced energy availability may activate brain-derived neurotrophic factor (BDNF) signaling, which is involved in synaptic plasticity and neurogenesis and may support adaptive brain responses to energetic challenges.

However, human studies have not provided clear evidence for a role of BDNF in the psychological changes observed during prolonged fasting [44,45]. Skurvydas et al. [46] reported improved mood, including increased vigor and reduced tension, during a 5 day fast, without significant changes in BDNF concentrations. These findings indicate that changes in self-reported mood during prolonged fasting need not occur in parallel with changes in this biomarker.

In an earlier publication based on the same participants and the same 8-day fasting experiment, the authors also reported a significant improvement in body image after the intervention [33]. These changes, together with the neurobiological processes accompanying metabolic adaptation, may have contributed to the observed improvement in subjective well-being.

### 4.2. Neuroendocrine Changes

In this study, changes in hormone concentrations were examined in the context of two physiological stimuli: an 8-day fast and maximal exercise. The most pronounced changes were observed in the post-fasting, post-exercise condition.

Detailed analysis showed that the largest increase in serotonin concentrations occurred in the post-fasting, post-exercise condition. Serotonin concentrations were significantly higher in the post-fasting, post-exercise condition than in the corresponding post-fasting resting condition. These findings indicate that serotonergic activation was most pronounced in the post-fasting, post-exercise condition. Kocahan et al. [18] reported similar observations, with increased serotonin and melatonin concentrations after exercise. Şentürk et al. [17] described increased serotonin concentrations after Ramadan fasting, accompanied by a greater subjective sense of happiness. This appears consistent with serotonin’s physiological role as a major neurotransmitter regulating mood, cognitive function, and gut–brain axis activity. However, previous studies indicate that the serotonergic response to exercise is not consistent. Hicks et al. [19] observed no changes in serotonin concentrations in athletes experiencing a runner’s high, and Filonzi et al. [47] found no association between serotonergic function and athletic performance. Carvalho Silva et al. [21] also noted that the effects of exercise on the monoaminergic system in individuals with severe depression are limited and variable. Regarding fasting, more than 90% of serotonin is produced in the gastrointestinal tract; prolonged restriction of food intake may therefore alter serotonergic function. This is supported by Haleem’s observations [48] of reduced serotonin concentrations in individuals with anorexia nervosa and by Gauthier et al. [49], who described an association between reduced tryptophan availability and more severe depressive and anxiety symptoms and impaired behavioral control. Together, these findings indicate that serotonergic activity may vary across conditions involving fasting and exercise.

A similar pattern was observed for α-endorphin. The highest α-endorphin concentration was observed in the post-fasting, post-exercise condition, whereas the relevant comparisons involving the other conditions did not show significant differences. These findings are only partly consistent with earlier observations of opioid system activation during fasting [24,25]. Research has also shown that increased activity of the endogenous opioid system may support improvements in mood, neuroplasticity, and learning [37]. Reduced pain perception has been reported after 24 h of food deprivation [50], whereas Komaki et al. [51] found that increased endorphin concentrations after a 10 day fast were not associated with the amount of weight lost. In the present study, α-endorphin concentrations did not differ significantly between the resting and post-exercise conditions before fasting. This observation differs in part from the findings of Viru and Tendzegolskis [52], who reported increased endorphin concentrations after exercise as well. The present study’s results therefore differ partly from reports of opioid system activation during fasting or following exercise. In the present study, increased serum α-endorphin concentrations were observed in the post-fasting, post-exercise condition.

The highest cortisol concentrations were observed in the post-fasting, post-exercise condition. Post-exercise cortisol concentrations were significantly higher after fasting than before fasting, whereas no significant difference was observed between the resting and post-exercise conditions before fasting. These findings are consistent with earlier studies showing that fasting activates the HPA axis and increases cortisol secretion [53,54]. Nakamura et al. [31] also showed that cortisol concentrations peak early in fasting and then gradually decline as metabolic adaptation develops. A similar adaptation has been observed during prolonged exercise training programs, in which basal cortisol concentrations gradually decreased despite repeated exercise demands [55].

No significant changes in oxytocin concentrations were found. This differs from the findings of Schroeder et al. [56], who reported reduced oxytocin concentrations only after 90 days of caloric restriction in rats, and from the work of Szabó et al. [28], who described an exercise-induced increase in oxytocin concentrations. These discrepancies indicate that differences in oxytocin system activity across conditions involving fasting and exercise require further investigation.

### 4.3. Changes and Interrelationships Among the Variables Studied

Despite concurrent psychological and neuroendocrine changes, no significant relationships were found between changes in resting hormone concentrations and changes in well-being or mood. These findings indicate that the psychological response does not simply mirror changes in individual hormone concentrations, rather, results from the temporal coexistence of specific changes that depend on the interaction of multiple regulatory mechanisms. Increased cortisol concentrations were not accompanied by poorer well-being or increased TA. Instead, well-being gradually improved and TA decreased over time. This finding indicates that HPA axis activation during prolonged fasting need not imply greater subjectively experienced psychological stress. In this group, increased cortisol concentrations appear primarily to reflect physiological mobilization for adaptation to metabolic stress, consistent with cortisol’s central role in regulating the stress response and maintaining metabolic homeostasis [53].

Similarly, the concurrent increases in serotonin and α-endorphin concentrations showed no simple relationships with the observed improvement in well-being. One of the factors that may have influenced the absence of a correlation between circulating neurohormone concentrations and well-being and mood could be the relatively small group size in this study. The absence of significant correlations does not exclude the involvement of these systems in regulating the psychological response. Rather, it suggests that relationships between neuroendocrine activity and affective changes are multifactorial and cannot be explained by the action of a single hormone.

One possible explanation for the absence of simple relationships between neuroendocrine responses and changes in well-being and mood involves cognitive appraisal of the situation and the coping strategies used. According to the transactional theory of stress, the psychological response is not simply a consequence of the stressor but depends on ongoing cognitive appraisal of that stressor and of the resources available to cope with situational demands [57]. During prolonged fasting, appraisal may change as fasting continues and adaptation develops, potentially influencing subjective well-being and mood independently of the direction of physiological changes. Because cognitive appraisal of the stressor and coping strategies were not assessed in this study, this explanation remains hypothetical and requires evaluation in future research.

### 4.4. Proposed Hypothesis-Generating Model of Psychoneuroendocrine Adaptation

These findings provide a basis for proposing a hypothesis-generating model of psychoneuroendocrine adaptation to prolonged fasting. Reduced energy intake was accompanied by differences in HPA-axis-related parameters and circulating serotonin and α-endorphin concentrations across the examined experimental conditions. In this framework, the concurrent psychological and hormonal changes provide a basis for considering their potential interrelationships as a hypothesis requiring further investigation. The observed changes in circulating serotonin and α-endorphin concentrations may be considered as potential correlates of psychological adaptation; however, the present findings do not establish a direct relationship between these responses.

The findings suggest that prolonged fasting was accompanied by gradual psychological adaptation, expressed as improved well-being and reduced TA alongside the observed neuroendocrine changes.

The proposed hypothesis may also be considered from an evolutionary perspective. When food availability is limited, the body must mobilize the energy resources needed for survival while maintaining the capacity to engage in food-seeking activity. In this context, reduced subjectively experienced tension and maintained well-being could hypothetically facilitate functioning during periods of energy deficiency. The proposed hypothesis-generating model requires validation in studies with larger samples and measurements at successive stages of prolonged fasting.

### 4.5. Study Limitations

This study was based on a relatively small sample size. This reflected the stringent eligibility criteria, which required subjects to be in good health and to have had previous experience of both prolonged fasting and of engaging in maximal physical exercise. The need to recruit a homogeneous group that met all these criteria imposed a severe practical limitation on the ability of the authors to increase the sample size, while also having the effect of reducing between-person variability in the results. Given our small sample size, we focused heavily on effect sizes rather than relying solely on traditional *p*-values. A small cohort naturally limits the power to find subtle correlations. However, the large magnitude of the effect sizes we found—across both psychological and hormonal variables—shows that these patterns are meaningful. This justifies treating our work as a solid, hypothesis-generating model.

A second limitation concerns the ability to make legitimate generalizations from the results. Owing to the previously described selection criteria, and the fact that the two female subjects originally selected discontinued their participation, the findings cannot be generalized to women, to individuals undertaking this type of fasting for the first time, or to those unused to maximal exercising.

A third limitation relates to the prior experience of the volunteers participating in these studies regarding fasting interventions and intense physical exertion. These factors created heightened expectations and the potential for placebo effects, which could have led to significant changes in mood and well-being, independent of neurohormonal changes.

Future studies should include larger samples, involving subjects with varying levels of fasting and exercise experience, and also include women, as well as establish a control group that meets the experimental requirements, thus allowing the roles of sex, previous fasting and exercise exposure, and potential confounding variables to be assessed with regard to psychological and neuroendocrine adaptation.

## 5. Conclusions

Prolonged fasting for 8 days was associated with gradual improvement in subjective well-being and reduced TA, accompanied by concurrent activation of selected neuroendocrine systems. The largest changes in α-endorphin, serotonin, and cortisol concentrations were observed following the combination of fasting and exercise. Fasting was associated with a significant increase in post-exercise cortisol concentrations, whereas exercise after fasting was associated with a significant increase in serotonin concentrations.

Despite concurrent psychological and neuroendocrine changes, no consistent relationships were detected among the variables examined in this sample. These findings indicate that psychological and neuroendocrine changes occur in parallel during prolonged fasting, although the present study does not establish direct relationships between these responses.

The concurrent psychological and neuroendocrine changes observed in this study provide a basis for a hypothesis-generating model of adaptation to reduced energy intake. This model proposes potential relationships between these responses but does not establish a coordinated mechanism of adaptation. The proposed hypothesis-generating model requires further evaluation in studies with larger samples and measurements at successive stages of prolonged fasting.

## Figures and Tables

**Table 1:** Demographic and somatic variables (M ± SD) of volunteers before the 8-day fast.

	M ± SD	Minimum	Maximum	Range
Age yrs	59.8 ± 12.37	37	77	37–77
Height cm	178.133 ± 4.75	167.00	184.00	167–184
Body weight kg	88.240 ± 11.95	69.60	108.00	69.6–108.0
BMI	27.800 ± 3.43	22.10	34.10	22.1–34.1

**Table 2:** Fixed effects of time from linear mixed-effects models assessing changes in mood dimensions during the 8-day fasting period.

Outcome Variable	Estimate (β)	95% CI	t	*p*
Tense arousal (TA)	−0.229	−0.416 to −0.041	−2.42	0.017
Hedonic tone (HT)	−0.034	−0.173 to 0.104	−0.49	0.626
Energetic arousal (EA)	−0.003	−0.183 to 0.176	−0.04	0.972

Note. β = estimated fixed effect of time, representing the average change in the outcome per day; CI = confidence interval.

**Table 3:** Percentage distribution of well-being responses on each day.

Well-Being	I	II	III	IV	V	VI	VII	VIII
Worse than usual	40.00	46.67	13.33	33.33	33.33	26.67	33.33	13.33
Same as usual	40.00	20.00	53.33	20.00	26.67	20.00	26.67	26.67
Better than usual	20.00	33.33	33.33	46.67	40.00	53.33	40.00	60.00

**Table 4:** Comparison of well-being between the first and eighth days of the 8-day fast (Wilcoxon signed-rank test).

Comparison	Day I M ± SD	Day I Me (Q1–Q3)	Day VIII M ± SD	Day VIII Me (Q1–Q3)	Z	*p*	Effect Size (r)
I–VIII	1.800 ± 0.775	2 (1–2)	2.467 ± 0.743	3 (2–3)	2.344	0.019	0.741

Note. M = mean; SD = standard deviation; Me = median; Q1–Q3 = lower and upper quartiles; Z = standardized Wilcoxon signed-rank test statistic; r = effect size. The analysis included 10 pairs with nonzero differences; five participants reported identical well-being ratings on days I and VIII.

**Table 5:** Comparison of Tense Arousal (TA) scores between days I and VIII of the 8-day fasting period (Wilcoxon signed-rank test).

Comparison	Day I M ± SD	Day I Me (Q1–Q3)	Day VIII M ± SD	Day VIII Me (Q1–Q3)	Z	*p*	Effect Size (r)
I–VIII	20.000 ± 3.703	20 (17–22)	17.333 ± 2.289	17 (15–20)	2.551	0.011	0.707

Note. N = total number of participants; M = mean; SD = standard deviation; Me = median; Q1–Q3 = lower and upper quartiles; Z = standardized Wilcoxon signed-rank test statistic; r = effect size. Thirteen participants had nonzero paired differences and were included in the rank calculation; two participants had identical TA scores on days I and VIII.

**Table 6:** Changes in hormone concentrations measured at rest and after exercise, before and after an 8-day fast (M ± SD; median; lower and upper quartiles).

Marker	Rest Pre	Exercise Pre	Rest Post	Exercise Post	*p*	Effect Size (η^2^)
Alpha-endorphin (pg/mL)	457.193± 255.992	478.367 ± 266.043	518.814 ± 314.491	651.834 ± 314.778	0.002	0.139
	471.94 (15.63–891.5)	480.93 (34.62–946.45)	544.02 (34.36–995.44)	774.65 (75.94–996.76)		
Post hoc	Rest pre vs. Exercise post *p* < 0.001					
Oxytocin (pg/mL)	267.588 ± 213.428	269.379 ± 222.918	340.491 ± 331.881	214.086 ± 264.060	0.932	0.004
	228.97 (5.51–645.87)	360.18 (7.93–637.34)	227.6 (9.3–914.6)	70.5 (12.2–796.89)		
Post hoc	-					
Serotonin (5-HT, ng/mL)	134.865 ± 109.180	366.069 ± 211.449	250.515 ± 193.396	632.387 ± 237.548	<0.001	0.202
	88.6 (40.74–349.8)	372.17 (46.235–781.97)	268.41 (39.6–543.1)	743.78 (237.1–900)		
Post hoc	Rest pre vs. Exercise post *p* < 0.001; Rest post vs. Exercise post *p* = 0.028					
Cortisol (ng/mL)	295.580 ± 92.102	332.520 ± 111.623	468.633 ± 111.196	529.620 ± 108.852	<0.001	0.218
	321.6 (126.2–450.3)	347.9 (125.7–553.1)	448.4 (309.9–733.8)	511.8 (375.1–763.3)		
Post hoc	Rest pre vs. Exercise post *p* < 0.001; Exercise pre vs. Exercise post *p* = 0.004					

**Table 7:** Spearman correlations between psychological variables and hormonal measures before and after fasting, with FDR correction applied separately within each hormone.

**Measurements Before Fasting**					
**Hormone**	**Condition**	**Psychological Variable**	**ρ**	***p***	**q (FDR)**
Cortisol	Rest	TA	−0.544	0.036	0.231
Cortisol	Exercise	TA	−0.500	0.058	0.231
Cortisol	Exercise	EA	0.394	0.146	0.390
Serotonin	Exercise	Well-being	−0.713	0.003	0.023
Serotonin	Exercise	HT	−0.632	0.011	0.046
Serotonin	Exercise	TA	0.433	0.107	0.285
**Measurements After fasting**					
**Hormone**	**Condition**	**Psychological Variable**	**ρ**	***p***	**q (FDR)**
Serotonin	Rest	HT	−0.455	0.088	0.925
Serotonin	Exercise	Well-being	−0.594	0.019	0.312
Cortisol	Exercise	EA	0.443	0.099	0.573

**Table 8:** Spearman correlations between changes (Δ) in resting hormonal biomarkers and psychological variables.

Biomarker (Δ)	Psychological Variable (Δ)	ρ	*p*	*p* (FDR)	Biomarker (Δ)	Psychological Variable (Δ)	ρ	*p*	q (FDR)
Cortisol (Rest)	Well-being	−0.115	0.682	0.913	OT (Rest)	Well-being	0.098	0.728	0.913
Cortisol (Rest)	TA	−0.303	0.273	0.780	OT (Rest)	TA	0.114	0.685	0.913
Cortisol (Rest)	HT	0.267	0.335	0.780	OT (Rest)	HT	−0.108	0.701	0.913
Cortisol (Rest)	EA	0.381	0.161	0.780	OT (Rest)	EA	−0.007	0.980	0.980
AEP (Rest)	Well-being	−0.075	0.791	0.913	5-HT (Rest)	Well-being	−0.185	0.510	0.913
AEP (Rest)	TA	−0.091	0.748	0.913	5-HT (Rest)	TA	0.159	0.570	0.913
AEP (Rest)	HT	−0.052	0.853	0.913	5-HT (Rest)	HT	0.060	0.833	0.913
AEP (Rest)	EA	0.239	0.391	0.780	5-HT (Rest)	EA	0.354	0.195	0.780

## Data Availability

The datasets generated and analyzed during the current study are available from the corresponding author on reasonable request.
